# A New Perspective of Urban–Rural Differences: The Impact of Social Support on the Mental Health of the Older Adults: A Case from Shaanxi Province, China

**DOI:** 10.3390/healthcare9020112

**Published:** 2021-01-21

**Authors:** Chi Zhang, Sifeng Zhang, Qing Niu

**Affiliations:** School of Public Policy and Administration, Xi’an Jiaotong University, Xi’an 710049, China; zc499167736@stu.xjtu.edu.cn (C.Z.); niuqing0103@stu.xjtu.edu.cn (Q.N.)

**Keywords:** social support, older adults, mental health, urban–rural differences

## Abstract

With the increase in aging in China, the health problems of older adults, especially mental health problems, have become a concern for the whole society. This article selected urban and rural older adults and analyzed the impact of social support on their mental health using a binary logistic model. It was found that under the current urban–rural dichotomy, the effects of social support on the mental health of urban and rural older adults are significantly different. In social support, first, the fairness and satisfaction with the social security system only had a significant effect on the mental health of urban older adults and had no significant effect on the rural older adults. Second, the closeness of contact with grassroots community workers had a significant impact on the mental health of older adults in urban and rural areas. From informal social support, the mental health of rural older adults was mainly influenced by the support of their children, reflecting the influence of the traditional culture of “filial piety”. Furthermore, the mental health of urban older adults was mainly influenced by neighborhood support, reflecting the importance of “close neighbors are better than distant relatives”. Based on the results of the empirical study, this article suggests that to promote the mental health of older adults, we should start by strengthening the formal social support system, establishing high-quality community service facilities, and emphasizing the role of informal social support.

## 1. Introduction

The increasing number of older adults is a major issue facing Chinese society as it enters a new period, and an area of national concern that Chinese society will face in the future. At the end of 2019, China’s population aged 60 and above reached 253.88 million, accounting for 18.1% of the total population [1]. In the coming period, with the increase in aging, the health of older adults will have an important impact on the socio-economic development of the country for a considerable period. In recent years, to face the impact of the aging population, the government and various aspects of society have taken on many positive measures. However, compared to the growing needs for a better life for the whole society, especially the aging population, chronic diseases, major diseases, and other health problems are still the main factors affecting the security of older adults and even the public in society [2].

The World Health Organization (WHO) considers mental health an important component of overall health, and mental health is more important than physical health [3]. Mental health plays an important role in the quality of life in older adults [4]. At least 100,000 older adults over 55 commit suicide every year in China, which is the population with the highest suicide rate in China at present [5]. There are more serious mental health problems among older adults in China. How to improve the mental health and quality of life of older adults in their later years has become an important social issue.

The “National Medium-and-Long-term Plan for Actively Coping with the Aging of the Population”, published in November 2019, emphasizes that “It is necessary to improve the multi-level social security system to promote a healthy level of life for older adults in their later years”. The “Healthy China” strategy also makes new requirements for the health of older adults. As individuals continue to grow older, older adults will have certain changes in their physiological functions and mental activities. At the same time, there will also be changes in their living environment and interpersonal relationships, which affect the mental health of older adults to varying degrees [6]. Due to the long-standing urban–rural dual structure in China, older adults live in urban and rural areas. There are still great differences in resource allocation and social welfare between urban and rural older adults, especially in health insurance and pension insurance [7], which are closely related to the life of older adults [8]. In general, urban older adults have better pension income [9], health convenience, health care coverage, and living convenience than rural older adults in the most part, and urban older adults are significantly better off than rural seniors in terms of average life expectancy, habitat, and mortality from disease. All objective evidence points to a huge imbalance in access to social support for older adults in urban and rural areas.

Social support for older adults is a general term for all kinds of support from the outside [10]. Meanwhile, social support for older adults is an objectively existing social relationship. This social relationship is mainly generated between individuals and between individuals and organizations. From the perspective of the support subject, social support is divided into formal and informal social support. Social support is defined as material assistance, behavioral assistance, intimate communication behavior, and social communication [11]. Social support is divided into three categories: formal social support, church support, and informal social support. Social support will play a role in alleviating stress [12]. Additionally, the acquisition of family support improves the subjective well-being and mental health of older adults. 

From the perspective of research objects, most studies of older adults do not distinguish where they live and there is a lack of comparative research on older adults in urban and rural areas; in the research on the impact of formal support on the mental health of the older adults, most are covered by health insurance and pension insurance. Starting with the question of rate, there is a lack of perspective on satisfaction with health insurance and pension insurance. Generally speaking, there is a lack of comprehensive and systematic research on the mental health of older adults and the research perspective is relatively single and lacks research on the mental health of older adults from the perspective of urban–rural and welfare equity.

## 2. Hypothesis and Framework

Based on the previous research results [13,14], this study intends to further consider and improve the following three aspects. First, a number of scholars have studied the impact of social support on the mental health of specific groups of older people such as those living alone and those with disabilities [15], but have ignored a comprehensive consideration of the impact of social support on the mental health of older adults in different living environments from the perspective of urban and rural areas. Second, as it relates to formal support. Most scholars have only considered the availability of health insurance and pension insurance [16], but have ignored the satisfaction of health insurance and pension insurance [17]. At this stage, China has basically achieved comprehensive coverage of health insurance and pension insurance [18]. As demands from older adults for a better life continue to increase, the issue of satisfaction and fairness is more important. Therefore, when examining the impact of formal social support on the mental health of older adults, the satisfaction of health insurance and pension insurance should be included in the model. Third, among informal social support, more scholars have considered the influence of intergenerational support, or child support on the mental health of older adults [19], although a small number of scholars have begun to focus on the influence of social relationships such as marital relationships (husband–wife relationships) [20], community relationships, and friendships on the mental health of older adults [21]. However, considering the range of activities and social characteristics of our older adults as well as the spatial distance of their daily life and travel, the above-mentioned social support is still missing. The construction of communities in urban and rural areas has provided more opportunities for older adults to communicate with their neighbors and should be included in the analysis of the social relationships of older adults. Numerous studies have shown that there are two mechanisms of social support on mental health, namely, the main effect model and the buffer model [22]. The main effect model assumes that social support has an absolute gain, which mainly means that as long as social support increases, the individual’s health status is bound to improve, regardless of the individual’s current level of social support [23]; the buffer model suggests that social support can act as a buffer for mental health and can mitigate the effects of adverse factors on physical and mental health [24]. 

**Figure 1:**
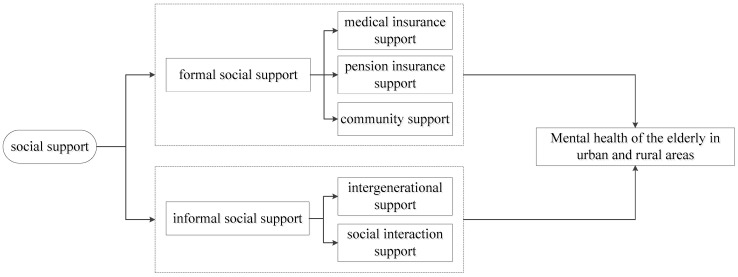
Research framework.

Ultimately, this study constructs a framework for analyzing the factors influencing social support on the mental health of urban and rural older adults, as detailed in Figure 1.

In summary, we believe that formal and informal support have a significant positive impact on their mental health, but there is a significant difference between urban and rural older adults in the effect of social support on their mental health, so the following hypothesis was proposed:

**Hypothesis 1** **(H1).** *Individual characteristics significantly affect and differ between urban and rural older adults’ mental health.*

From the perspective of individual characteristics, the happiness of marriage has a positive effect on the mental health of older adults [25]. Individual characteristics have an important impact on the well-being of older adults, and well-being is closely related to the mental health of older adults [26]. Different age groups and different personalities also have a significant impact on individual mental well-being [27].

**Hypothesis 2** **(H2).** *Formal social support has a significant effect on the mental health of urban and rural older adults.*

In formal support, health insurance and pension insurance significantly affect the health of the older adults [28]. The improvement of health insurance coverage and satisfaction can promote the improvement of the mental health of older adults [29]. Formal support can effectively alleviate the chronic diseases of older adults and improve the level of mental health [30]. The basic public health services implemented by the government will also promote the health of older adults [31]. The increase in pension income will significantly improve the mental health of older adults [32]. The increase in pension coverage will also improve the mental health of older adults [33].

**Hypothesis 3** **(H3).** *Informal social support has a significant effect on the mental health of urban and rural older adults*.

In terms of informal support, the habitat environment also has a significant impact on the mental health of older adults, particularly parks, walkability, and connectivity [34]. Furthermore, the improvement of social status will improve the mental health of older adults in their later years [35]. Intergenerational support is the most important social support for older adults. Emotional support and appropriate tools and financial support have a significantly positive impact on the mental health of older adults [36]. Family support, especially communication from their children, has dramatically improved the mental health of older adults [37]. In urban communities, the lack of social support for older adults will greatly increase the risk of depression [38]. Informal support provided by friends plays an important role in reducing depression [39].

**Hypothesis 4** **(H4).** *There is a significant difference between the effects of formal social support and informal social support on the mental health of urban and rural older adults.*

There are significant differences in mental health levels between urban and rural areas [40]. The mental health of older adults in urban China is generally higher than that of older adults in rural areas [41]. In terms of factors affecting mental health, urban older adults and rural older adults also show differences [42]. Compared with urban elders, the mental health of rural older adults depends more on the social field [43] and the support of their children [44].

## 3. Data and Method

### 3.1. Data Source

The data for this study were obtained from the field research of the subject team. We used a stratified random sampling method to conduct field research in Yan’an, Baoji, and Hanzhong cities in Shaanxi Province from July to August 2019. The survey team consisted of 10 teachers, eight PhD students, and 15 master students from Xi’an Jiaotong University. One hundred and seventeen respondents participated in the pre-study. The first stage of stratification was based on cities, the second stage of stratification was based on counties, the third stage was based on villages, and the fourth stage of stratified sampling was based on a sample of older adults. Three cities, Han Zhong, Baoji, and Yan’ an, were selected because they are representative areas characterized by high aging and the rapid development of mental health for older adults. Our initial aim was to collect 1000 samples. In the end, we obtained 948 valid questionnaires related to the theme, which is representative of the population. After the questionnaires were screened and eliminated, we used 670 questionnaires that finally matched our research theme.

### 3.2. Variable Selection

The dependent variable is the self-reported mental health of older adults. This was divided into two categories, where the first category is mental health or relatively healthy psychology. This was assigned a value of 1 to indicate that older adults were mentally healthy. Those who chose unhealthy or less healthy were assigned a value of 0, indicating that the older adults were mentally unhealthy. According to social support theory and previous studies, the independent variables are social support including formal social support and informal social support, the former includes health insurance support (availability of health insurance, health insurance satisfaction), pension insurance support (availability of pension insurance, pension insurance satisfaction), community support (frequency of contact with community members), and the latter includes intergenerational support (mode of residence, frequency of children’s communication, frequency of financial support from children), and social interaction (frequency of helping each other with neighborhood friends, frequency of communicating with neighborhood friends). The control variables mainly include age, gender, marital status, urban–rural distribution, education level, political outlook, and personal income. In summary, the variable of this paper is representative, and the specific sample characteristics are shown in Table 1.

### 3.3. Analysis Method

With the help of SPSS 24.0 statistical software (IBM, Armonk, NY, USA), a descriptive analysis of control variables and social support was carried out, and logistic regression analysis was used to explore the factors affecting the mental health of older adults in urban and rural areas. The dependent variable mental health (Y) is a binary variable. When the mental health of urban and rural older adults is healthy, Y = 1; otherwise, Y = 0.

### 3.4. Describing Sample

Of the all participants in the survey, 43.9% of the older adults were aged 60–69 and 34.2% of the older adults were aged 70–79. A total of 21.9% of the older adults were aged 80 years or older, with an overall balance aged distribution of the older adults. Men accounted for 42.7% and women accounted for 57.3%. The proportion of education level was 48% for elementary school and below, 26.3% for junior high school, 18.0% for high school, and 7.7% for college and above. In terms of marriage, the proportion of urban older adults with a spouse was 56.5%, the proportion of rural older adults with a spouse was 43.5%, and the proportion of older adults without a spouse was larger. A total of 54.6% of the older adults lived in the city, and 45.4% of the older adults lived in the countryside, and 130 older adults lived alone, accounting for 13.7%, indicating that a considerable number of elderly people still lived alone. In terms of the relationship between the older adults and their children, 77.2% of the older adults and their children often communicated with each other, and 78.6% of the older adults and their children often supported each other (economic mutual assistance and sharing of household chores). Regarding the relationship between the older adults and their neighbors and friends, 82.0% of the older adults often helped each other and 85.7% of the older adults often had activities and chatted with their neighbors and friends. The percentage of seniors who participated in health insurance was 96.6%, and the percentage of seniors who participated in pension insurance was 93.6%, indicating that health insurance and pension insurance in China have basically reached full coverage among seniors. However, the satisfaction rate of health insurance was 61.5%, and the satisfaction rate of the pension insurance was 52.6%, and 38.5% and 47.4% of the older adults were dissatisfied with health insurance and pension insurance, respectively. In summary, the sample of this paper is representative, and the specific sample characteristics are shown in Table 2.

In terms of income structure, among urban residents, 71.19% said that the pension (or retirement pension) was their main source of income; followed by children’s support, accounting for 37.01% of the total combined trips, and the main source of livelihood for rural groups was also concentrated in these two parts, with the pension being the main source of livelihood for the largest number of trips, followed by children’s support, accounting for 47.16% and 20.45%, respectively. Table 3 shows that pensions have now become the main source of income for urban and rural older adults.

## 4. Results

### 4.1. Regression Analysis

We took the mental health of older adults as the dependent variable, social support (formal social support, informal social support) as the independent variable, and gender, age, marriage, education level, political status, and income as the control variables [45,46,47,48]. Binary logistic regression analysis was conducted for the urban and rural elderly. Model 1 incorporated only control variables, and model 2 added social support variables to the control variables. Model 1 incorporated control variables only, model 2 added formal social support variables on top of control variables, and model 3 was a full model, incorporating control variables, formal social support variables, and informal social support variables. Table 4 and Table 5 show the regression results for the urban older adults and the rural older adults.

As shown in Table 4 and Table 5, only education level among the control variables of model 1a had a significant effect on mental health and remained significant in models 2a and 3a, and marital status and education level among the control variables of model 1b had a significant effect on mental health, which remained robust in models 2b and 3b. To test hypothesis 1, individual characteristics had a significant effect on the mental health of urban and rural older adults.

In model 2a, frequency of contact with community members, satisfaction with health insurance, and satisfaction with pension insurance had significant effects on the mental health of urban older adults, and this result remained robust in the full model 3a. In model 3a, frequency of contact with community members, satisfaction with health insurance, and satisfaction with pension insurance were significant. In model 2b, the effect of frequency of contact with village committee personnel on the mental health of rural older adults was significant, and the result remained robust in model 3b. In model 3b, the effect of frequency of contact with village committee staff on the mental health of rural older adults was significant. Thus, when testing hypothesis 2, formal social support had significant effects on the mental health of urban and rural older adults.

In model 3a, frequency of children communication, frequency of neighborhood friends helping each other, and frequency of neighborhood friends communicating with each other had significant effects on the mental health of urban older adults, and in model 3b, frequency with children communication, financial support from children, and frequency of neighborhood friends helping each other had significant effects on the mental health of rural older adults. Thus, when testing hypothesis 3, informal social support had significant effects on the mental health of urban and rural older adults.

In summary, due to the differences in the influence factors of social support for mental health between urban and older adults, we also verified hypothesis 4, where there was a significant difference between the effects of formal social support and informal support on the mental health of urban and rural older adults.

### 4.2. Analysis of Results

It can be seen from the above regression results, in terms of control variables, that the effects of gender and age on mental health were not significant in urban older adults, indicating that gender and age could not significantly affect the mental health of urban older adults. Marital status did not have a significant effect on mental health, indicating that the presence or absence of a spouse did not have a significant effect on mental health because older adults without a spouse received more care from their children and community, in all probability, which compensated for the sense of loss without a spouse, so it could be assumed that the presence or absence of spouse status did not affect the self-rated mental health of older adults. Educational level considerably influenced the mental health of urban older adults, and the probability of urban older adults with higher educational level having a good mental health status increased by 72.8%. Political status did not have a significant impact on the mental health of urban older adults. In rural older adults, marital status had a significant effect on the mental health of older adults, and the probability of a good mental health status of older adults with a happy marriage increased by 37.5%. Educated older adults had a significant impact on the mental health of older adults, and the probability of a good mental health status of older adults with high education level increased by 32.4%. Rural older adults with a high education level had a stronger ability to overcome difficulties in life due to their high knowledge and culture level, which was also conducive to continuous self-affirmation, more accurate cognition of society, and a perfect social network, thus having better mental health. Other factors such as gender, age, and political status did not have a significant impact on the mental health of rural older adults.

From the perspective of formal social support, the frequency of contact with community staff had a significant effect on the mental well-being of older adults in urban older adults, with the probability of mental well-being increased by 99.2% (OR 1.992) among those who had frequent contact with community staff. Satisfaction with health insurance had a significant effect on the mental health of older adults, with the probability of being satisfied with health insurance increased by 79.9% (OR = 1.797), and satisfaction with pension insurance had a significant effect on the mental health of older adults, with the probability of being satisfied with pension insurance increased by 223% (OR = 2.233). Regardless of how, the presence or absence of health insurance and pension insurance did not have a remarkable impact on the mental health of older adults, combined with the reality of China’s health insurance and pension insurance, which has basically achieved a bottom-up coverage for all older adults. In contrast, urban older adults were more concerned about the quality of services provided by health insurance and old-age pension insurance, which solved their daily health expenses due to the increase of disease incidence in older adults, and higher pension income and better health insurance services significantly improved the mental health of urban older adults. Furthermore, from the perspective of informal social support, the probability of having good mental health status of older adults who had frequent contact with their children was 216% higher than those who had infrequent contact (OR = 2.169). The probability of having good mental health status of older adults who frequently helped each other with neighboring older adults increased by 206% (OR = 2.057) than those who did not frequently help each other. The probability of good mental health level of older adults who communicated economically with other older adults was 11% higher than those who did not communicate frequently (OR = 1.114). Older adults gained more positive emotions such as pleasure and satisfaction from communicating and helping each other within the group, and good social relationships played an important role in improving the life satisfaction and mental health of older adults.

From the perspective of formal social support, among the rural older adults, the frequency of contact with village committee cadres had an important influence on the mental health of older adults, and the probability of having good mental health status of older adults with close contact with village cadres was 257% higher than that of the older adults with less close contact with village cadres (OR = 2.586). Most of the rural older adults had children who were away from home, so village cadres played a particularly important role in caring for older adults in their daily lives, solving their difficulties in time and chatting with them, which effectively improved the mental health of the rural older adults. From the perspective of informal social support, the probability of having good mental health status of older adults with financial support from their children was 80% higher than that of the older adults without financial support from their children (OR = 1.813). The probability of having good mental health status of older adults with frequent contact with their children was 311% higher than that of older adults with infrequent contact with their children (OR = 3.113). Combined with the reality of our country, children’s financial support and communication had a pivotal role in the mental health of rural older adults, which also reflected the filial piety of the children to older adults, so that the rural older adults had better mental health. Older people who stayed in rural areas obtained more positive emotions such as pleasure and satisfaction through the process of communication with their children, thus promoting their mental health. Health insurance, pension insurance, and living style did not significantly affect the mental health of the older adults, and the reality was that, at present, rural older adults generally had lower levels of material demand. Therefore, objective material conditions did not have a significant impact on the mental health of the older adults.

### 4.3. Robust Test

In order to test the robustness of this article, we further selected another question “Do you feel lonely and isolated?” in the questionnaire as the measurement variable of mental health. The test was re-run according to the new dependent variables. Table 6 shows the results of the robust test.

The results in Table 6 show that education had a significant effect on the mental health of urban older adults, and marriage and education had a significant impact on the mental health of rural older adults. Hypothesis 1 of this article was still validated. Frequency of contact with government staff, health insurance satisfaction, and pension insurance satisfaction had a significant impact on the mental health of urban adults, and frequency of contact with government staff had a significant impact on the mental health of rural elderly, thus hypothesis 2 of this article was still validated. Children’s communication frequency, frequency of helping each other with neighbors, and frequency of communication with neighbors had a significant impact on urban older adults mental health. Children’s communication frequency, children’s financial support, and frequency of helping each other with neighbors had a significant effect on the mental health impact of rural adults. Hypothesis 3 of this article was still validated. Finally, hypothesis 4 of this article was still validated because there was a significant difference between formal and informal support on the mental health of rural and urban adults. Therefore, the findings of this article are robust.

## 5. Discussion

This paper constructed an analytical framework to explore the influence of social support on the mental health of urban and rural older adults. By comparing and analyzing the difference between formal and informal social support received by urban and rural older adults, we obtained the mechanism of the different influence of social support on the mental health of urban and rural older adults.

First, the level of education has a significant effect on the mental health of urban and rural older adults. The increase in the level of education helps residents to improve their perception of self as well as the impact of self-confidence and other effects of education on human character and mentality, all of which help to reduce the risk of mental disease. The level of education and mental health showed a positive correlation development [49].

There is no evidence to show that there was a significant difference between the mental health effects of having pension insurance and health insurance on the urban and rural older adults, and the urban older adults were more concerned about the level of health insurance and pension insurance benefits [50]. There are two main reasons for these results: first, China has basically achieved full coverage of health insurance and pension insurance; and second, the difference between the pension insurance and medical insurance systems in urban and rural areas has led to the inequality of social welfare for older adults in urban and rural areas. Since rural older adults in the same area currently receive similar levels of pension insurance and health insurance for basically every household, the issue of equity has been effectively addressed, therefore rural older adults do not have a significant effect on the availability of the two types of insurance. At the same time, the health needs and quality of old-age care of urban older adults are significantly higher than those of rural older adults [51], and the characteristics of China’s overall old-age security are “basic protection” and “wide coverage”. Therefore, urban older adults paid more attention to the supply level and quality of pension insurance and health insurance. 

Among the formal social support, the satisfaction of urban older adults with health insurance and pension insurance had a significant impact on their mental health. On one hand, with the increasing level of urbanization in China and the continuous development of the urban economy, the needs of urban older adults for a better life are rising. On the other hand, because of the urban–rural divide, rural areas are far less developed than urban areas, and the material needs of rural seniors are lower. The satisfaction of health insurance and pension insurance has contributed to the mental health of urban older adults much more than that of rural older adults. The pension insurance for urban older adults has basically achieved full coverage, but there is still a demand to improve the specific amount of pension. Gradually increasing the level of pension treatment is conducive to the improvement of the living standard of older adults, thus promoting their mental health. Compared with the health insurance for rural residents, urban health insurance has a higher coverage and reimbursement ratio, which can encourage urban older adults to go to the hospital more quickly to get treatment when they suffer from diseases in daily life, and to make full use of the convenient health services in the city at the subjective level. Therefore, health insurance satisfaction has a significant effect on the mental health of older adults in urban areas.

There is a noteworthy difference in the influence of informal social support on the mental health of urban and rural older adults. Frequent communication with children and frequent financial support from children can significantly improve the mental health of rural older adults, mainly due to the influence of “filial piety” in traditional Chinese culture, which is more obvious in rural areas [52]. At the same time, mutual communication and support among neighbors and friends can significantly improve the mental health of urban older adults because of the stronger social desire of urban older adults, which reflects the social phenomenon that distant relatives are not better than close neighbors among urban residents. Specifically, communication with children and the economic support provided by children can provide older adults in rural areas with certain emotional comfort, thus enhancing the mental health of the older adult group. The intergenerational support received by older adults from their children reflects the influence of filial piety in traditional Chinese culture, which makes the older adults feel the care and support of their children from the inside and enhances the sense of achievement and access to rural older adults in their later years. At the same time, intergenerational support provides a certain degree of livelihood security and support for the rural older adults. With both material and spiritual support, the sense of control and role in the life of older adults is gradually reinforced, which makes them feel their own value and thus enhances their mental health. Since urban older adults have higher pensions and retirement benefits than rural older adults, they have sufficient livelihood security and are less dependent on intergenerational economic support than rural older adults, so urban older adults need more spiritual comfort and communication. Most of the urban older adults are only children, and the social support provided by neighbors is more important because of the absence of children. This is in line with the social phenomenon of “distant relatives are not better than close neighbors”, and the social support provided by neighbors becomes an important factor that significantly affects the mental health of urban older adults.

In general, the mental health of both urban and rural older adults has a significant correlation with the formal support and informality to varying degrees. Providing adequate social support to older adults will promote the continuous improvement of the mental health of both urban and rural older adults, and the relationship between the two is mutually reinforcing; conversely, if urban and rural older adults receive lower levels of social support, it will pose a serious threat to the mental health of both urban and rural older adults.

The present article has some limitations such as the data limitation; the current article only studied the influence of social support on the mental health of urban and rural older adults in Shaanxi Province, but Shaanxi is a western province of China, which can only represent the western region of China, and it is debatable whether Shaanxi Province is representative for the eastern region of China. The future study can work on two aspects: (1) further expand the scope of the study as well as field research in developed eastern regions to verify and enrich the existing study; and (2) in the selection of social support-related indicators, the indicators can be adjusted and improved continuously through more detailed interview research in the future to provide a theoretical basis for enriching the analytical framework of the influence of social support on the mental health of urban and rural older adults. The main strength of this study is that it obtained first-hand data through field research, and distinguished formal support from informal support, and analyzed the differences affecting the psychological health of older adults from the perspective of social support, and the research content and research perspective are somewhat innovative.

## 6. Conclusions

Based on the data of 948 surveys obtained from a field survey in Shaanxi Province in 2019, this article selected urban and rural older adults aged 60 years and above in Shaanxi Province and analyzed the impact of social support on the mental health of urban and rural older adults using a binary logistic model. The current urban–rural was found, and the effects of social support on the mental health of urban and rural older adults were significantly different. 

The article comes to the following conclusions. Regarding formal social support, (1) fairness and satisfaction with the social security system only had a significant effect on the mental health of urban older adults and had no significant effect on rural older adults. (2) The closeness of contact with grassroots community workers significantly influenced the mental health of rural and urban older adults in terms of informal social support. (3) The mental health of rural older adults was mainly influenced by the support of their children, reflecting the influence of the traditional culture of “filial piety”. (4) The mental health of urban older adults was mainly influenced by neighborhood support, reflecting the importance of the saying “close neighbors are better than distant relatives”.

The article suggests improving the level and quality of formal and informal support for older adults. First, accelerate the construction of the formal social support system. The first task of the construction of a formal social support system is to establish a pension insurance and health insurance system that can cover all citizens and the bottom line should be ensured, and therefore the fruits of social development can be shared by all people. Due to the existence of the dual system of urban and rural areas, there is a great difference between older people in urban and rural areas in China in terms of pension insurance and health insurance, while health insurance also shows differences and unevenness in terms of releasing health needs. Second, establish comprehensive community support and services and increased investment in urban and rural community infrastructure. The establishment of diversified and specialized old-age service facilities should be popularized. Community counseling rooms should be built in the communities. Meanwhile, older adults are encouraged to contract family doctors, and doctors with professional mental counseling skills can communicate and exchange ideas with older adults on a regular basis to promote a good and positive attitude toward life and to facilitate the continuous improvement of their mental health.

## Figures and Tables

**Table 1:** Influencing factors of mental health of the older adults in urban and rural areas.

Variables	Name	Meaning
**Dependent variable**	Mental health	1 = Health, 0 = Unhealthy
**Control variable**	Gender	1 = Male, 0 = Female
	Age	1 = over 75 years old, 0 = 60–75 years old
	Marriage	1 = Married, 0 = Unmarried
	Registered permanent residence	1 = City, 0 = Countryside
	Education	1 = Primary school, 0 = Others
	Political status	1 = CPC, 0 = Others
	Income	Distance variable
**Formal social support**	Health insurance	1 = Have, 0 = No
	Pension	1 = Have, 0 = No
	Frequency of contact with government staff	1 = High, 0 = Low
	Health insurance satisfaction	1 = Satisfied, 0 = Dissatisfied
	Pension insurance satisfaction	1 = Satisfied, 0 = Dissatisfied
**Informal social support**	Way of living	1 = Live alone, 0 = Not living alone
	Children’s communication frequency	1 = High, 0 = Low
	Children’s financial support	1 = High, 0 = Low
	Frequency of helping each other with neighbors	1 = High, 0 = Low
	Frequency of communication with neighbors	1 = High, 0 = Low

**Table 2:** Basic characteristics of the sample.

Variables	Classification	Frequency	Percent (%)
Gender	Male	405	42.7
	Female	543	57.3
Age	60–69	414	43.9
	70–79	323	34.2
	80–89	188	19.9
	>90	19	2.0
Marriage	Married	536	56.5
	Unmarried	412	43.5
Education	Primary school	455	48.0
	Junior high school	248	26.3
	High school	170	18.0
	University	73	7.7
Political status	Party of the CPC	247	26.1
	Others	701	73.9
Registered permanent residence	City	515	54.6
	Countryside	429	45.4
Children’s communication frequency	High	731	77.2
	Low	217	22.8
Children’s financial support	High	745	78.6
	Low	203	21.4
Frequency of helping each other with neighbors	High	812	85.7
	Low	136	14.3
Frequency of communication with neighbors	High	777	82
	Low	171	18
Pension insurance coverage rate		887	93.6
Health insurance coverage		915	96.6
Pension insurance satisfaction		498	52.6
Health insurance satisfaction		583	61.5

**Table 3:** The main sources of living of the surveyed residents.

Sources of Income					
Registered Permanent Residence	Children Provide	Pension	Savings	Commercial Pension Insurance	Others
Countryside	20.45%	47.16%	10.45%	1.34%	3.88%
City	37.01%	71.19%	15.67%	0.75%	1.64%

**Table 4:** Logistic regression analysis of the mental health of urban older adults.

Control Variable	Model 1a OR (95% CI)	Model 2a OR (95% CI)	Model 3a OR (95% CI)
Gender	1.396 (0.925, 1.867)	0.844 (0.635, 1.053)	1.277 (0.916, 1.638)
Age	1.146 (0.815, 1.477)	1.014 (0.846, 1.182)	1.118 (0.887, 1.349)
Marriage	0.905 (0.784, 1.026)	1.082 (0.875, 1.289)	1.009 (0.805, 1.213)
Education	1.104 ** (1.012, 1.196)	1.572 ** (1.115, 2.029)	1.728 ** (1.346, 2.110)
Political status	1.385 (0.901, 1.869)	1.112 (0.945, 1.279)	0.962 (0.798, 1.126)
**Formal social support**			
Health insurance		0.988 (0.832, 1.144)	1.032 (0.811, 1.253)
Pension		0.819 (0.615, 1.023)	0.939 (0.785, 1.093)
Frequency of contact with government staff		1.357 *** (1.115, 1.599)	1.992 *** (1.747, 2.237)
Health insurance satisfaction		1.509 *** (1.047, 1.971)	1.797 *** (1.534, 2.060)
Pension insurance satisfaction		1.494 *** (1.028, 1.960)	2.233 *** (2.045, 2.421)
**Informal social support**			
Way of living			1.695 (0.801, 2.589)
Children’s communication frequency			2.169 * (1.928, 2.410)
Children’s financial support			1.037 (0.818, 1.256)
Frequency of helping each other with neighbors			2.057 *** (1.785, 2.329)
Frequency of communication with neighbors			1.114 *** (1.049, 1.179)

Note: *** means significant at the 1% level, ** means significant at the 5% level, and * means significant at the 10% level.

**Table 5:** Logistic regression analysis of mental health of rural older adults.

Control Variable	Model 1b OR (95% CI)	Model 2b OR (95% CI)	Model 3b OR (95% CI)
Gender	1.688 (0.823, 3.146)	1.365 (1.112, 1.618)	1.547 (0.984, 2.110)
Age	0.856 (0.581, 1.131)	0.858 (0.641, 1.075)	0.871 (0.788, 1.054)
Marriage	0.912 ** (0.742, 1.082)	1.165 ** (1.093, 1.237)	1.375 ** (1.145, 1.605)
Education	1.330 *** (1.124, 1.536)	1.193 *** (0.985, 1.401)	1.324 *** (1.126, 1.522)
Political status	1.503 (1.257, 2.131)	1.208 (0.921, 1.495)	1.244 (0.985, 1.503)
**Formal social support**			
Health insurance		1.125 (0.906, 1.344)	1.025 (0.944, 1.106)
Pension		0.871 (0.638, 1.104)	0.622 (0.218, 1.026)
Frequency of contact with government staff		1.982 ** (1.581, 2.383)	2.586 ** (2.156, 3.016)
Health insurance satisfaction		1.265 (0.996, 1.534)	1.377 (1.201, 1.863)
Pension insurance satisfaction		1.983 (0.894, 3.072)	2.199 (1.818, 2.580)
**Informal social support**			
Way of living			1.984 (1.784, 3.084)
Children’s communication frequency			3.113 *** (2.779, 3.447)
Children’s financial support			1.813 *** (1.568, 2.058)
Frequency of helping each other with neighbors			4.347 * (4.116, 4.578)
Frequency of communication with neighbors			0.973 (0.808, 1.138)

Note: *** means significant at the 1% level, ** means significant at the 5% level, and * means significant at the 10% level.

**Table 6:** The results of the robust test.

Variable	Model 1a OR (95% CI)	Model 2a OR (95% CI)	Model 3a OR (95% CI)	Model 1b OR (95% CI)	Model 2b OR (95% CI)	Model 3b OR (95% CI)
	Urban			Rural		
**Control Variable**						
Gender	1.125 (0.881, 1.369)	0.821 (0.613, 1.029)	1.384 (0.976, 1.792)	1.536 (0.889, 2.183)	1.425 (0.925, 1.925)	1.452 (0.936, 1.968)
Age	1.047 (0.836, 1.231)	1.115 (0.844, 1.386)	1.256 (0.894, 1.618)	0.872 (0.745, 1.093)	0.896 (0.584, 1.208)	0.912 (0.803, 1.021)
Marriage	0.925 (0.815, 1.035)	1.762 (0.983, 2.541)	1.009 (0.928, 1.090)	0.984 ** (0.863, 1.105)	1.172 ** (1.042, 1.302)	1.465** (1.162, 1.768)
Education	1.108 ** (1.071, 1.145)	1.717 ** (1.321, 2.113)	1.816 ** (1.536, 2.096)	1.325 *** (1.021, 1.629)	1.996 *** (1.681, 2.311)	1.514 *** (1.265, 1.763)
Political status	1.148 (0.907, 1.389)	1.135 (0.901, 1.369)	0.889 (0.761, 1.017)	1.471 (0.861, 2.081)	1.384 (0.954, 1.814)	1.342 (0.911, 1.773)
**Formal social support**						
Health insurance		0.991 (0.832, 1.150)	1.112 (0.874, 1.346)		1.145 (0.921, 1.369)	1.127 (0.908, 1.346)
Pension		0.845 (0.731, 1.059)	0.979 (0.831, 1.127)		0.752 (0.418, 1.086)	0.735 (0.388, 1.082)
Frequency of contact with government staff		1.148 *** (0.906, 1.390)	1.856 *** (1.614, 2.098)		1.981 ** (1.281, 2.681)	2.687 ** (2.447, 2.927)
Health insurance satisfaction		1.618 *** (1.284, 1.952)	1.825 *** (1.593, 2.057)		1.238 (0.853, 1.623)	1.389 (0.873, 1.905)
Pension insurance satisfaction		1.543 *** (1.195, 1.891)	2.112 *** (1.884, 2.340)		1.894 (0.903, 2.885)	2.129 (0.886, 3.372)
**Informal social support**						
Way of living			1.756 (1.414, 2.098)			1.684 (0.744, 2.624)
Children’s communication frequency			2.356 * (2.142, 2.570)			3.256 *** (2.889, 3.623)
Children’s financial support			1.365 (0.865, 1.865)			1.012 *** (1.002, 1.022)
Frequency of helping each other with neighbors			2.042 *** (1.691, 2.393)			4.347 * (3.988, 4.706)
Frequency of communication with neighbors			1.225 *** (0.987, 1.463)			0.896 (0.654, 138)

Note: *** means significant at the 1% level, ** means significant at the 5% level, and * means significant at the 10% level.

## References

[1] National Bureau of Statistics Population Age Structure in China 2019. https://data.stats.gov.cn/easyquery.htm?cn=C01.

[2] Zhou M., Wang H., Zeng X., Yin P., Liang X.F. (2019). Mortality, Morbidity, and Risk Factors in China and Its Provinces, 1990–2017: A Systematic Analysis for the Global Burden of Disease Study 2017. Lancet.

[3] Grad F.P. (2002). The preamble of the constitution of the World Health Organization. Bull. World Health Organ..

[4] WHO World Report on Ageing and Health. https://apps.who.int/iris/handle/10665/186463.

[5] Nie J.B. (2016). Erosion of eldercare in china: A socio-ethical inquiry in aging, elderly suicide and the government’s responsibilities in the context of the one-child policy. Aging Int..

[6] Liu Y., Dijst M., Faber J., Geertman S., Cui C. (2017). Healthy urban living: Residential environment and health of older adults in shanghai. Health Place.

[7] Lako C.J., Cluitmans R., Fredrix L., Vasbinder L. (1988). Health status and health consumption of the elderly in urban and rural areas in the netherlands: A secondary analysis of cbs-data. Z. Alternsforsch..

[8] Wang Z.W., Chen Y.C., Pan T.Y., Liu X.D., Hu H.W. (2019). The comparison of healthcare utilization inequity between URRBMI and NCMS in rural China. Int. J. Equity Health.

[9] Mckenzie K., Murray A., Booth T. (2013). Do urban environments increase the risk of anxiety, depression and psychosis? An epidemiological study. J. Affect. Disord..

[10] Rook K.S., Ituarte P.H.G. (1999). Social control, social support, and companionship in older adults’ family relationships and friendships. Pers. Relatsh..

[11] Barrera M. (1986). Distinctions between social support concepts, measures, and models. Am. J. Community Psychol..

[12] Card D., Dobkin C., Maestas N. (2019). Does Medicare Save Lives?. Q. J. Econ..

[13] Kikuzawa S. (2016). Social support and the mental health of family caregivers: Sons and daughters caring for aging parents in japan. Int. J. Jpn. Sociol..

[14] Wang L.F., Shi Y.J. (2008). Social Support and Mental Health of the Empty-nest Elderly People in Urban Area. Chin. Mental Health J..

[15] Jun E., Liu J.Y., Tian P., Du X.P. (2015). Living experience and care needs of Chinese empty-nest elderly people in urban communities in Beijing, China: A qualitative study. Int. J. Nurs. Sci..

[16] Yang H.Y., Ma J., Hu H.W., Li F.J. (2020). Identification, Trend Analysis and Influencing Factors of Mental Health Status of the Chinese Older Adults. Int. J. Environ. Health Res..

[17] Gao X., Feng T.Y. (2020). Public Pension, Labor Force Participation, and Depressive Symptoms across Gender among Older Adults in Rural China: A Moderated Mediation Analysis. Int. J. Environ. Health Res..

[18] Qi S.W., Zhou S.F. (2010). The Influence of Income, Health and Medicare Insurance on the Happiness of the Elderly in China. J. Public Manag..

[19] Liu D.X., Xi J., Hall B.J., Fu M.Q., Zhang B., Guo J., Feng X.L. (2020). Attitudes toward aging, social support and depression among older adults: Difference by urban and rural areas in China. J. Affect. Disord..

[20] Xie Y.F., Ma M.D., Wu W.W., Zhang Y.P., Zhang Y.T., Tan X.D. (2020). Dose-response relationship between intergenerational contact frequency and depressive symptoms amongst elderly Chinese parents: A cross-sectional study. BMC Geriatr..

[21] Amegbor P.M., Braimah J.A., Adjaye-Gbewonyo D., Rosenberg M.W., Sabel C.E. (2020). Effect of cognitive and structural social capital on depression among older adults in Ghana: A multilevel cross-sectional analysis. Arch. Gerontol. Geriatr..

[22] Rodriguez N., Flores R.T., London E.F., Mira C.B., Myers H.F., Arroyo D., Rangel A. (2019). A Test of the Main-Effects, Stress-Buffering, Stress-Exacerbation, and Joint-Effects Models Among Mexican-Origin Adults. J. Latinx Psychol..

[23] Beeble M.L., Bybee D., Sullivan C.M., Adams A.E. (2009). Main, Mediating, and Moderating Effects of Social Support on the Well-Being of Survivors of Intimate Partner Violence Across 2 Years. J. Consult. Clin. Psychol..

[24] Toyama M., Fuller H.R. (2019). Longitudinal Stress-Buffering Effects of Social Integration for Late-Life Functional Health. Int. J. Aging Hum. Dev..

[25] Kawachi I., Berkman L.F. (2001). Social ties and mental health. J. Urban Health.

[26] Beekman A.T.F., Penninx B.W.J.H., Deeg D.J.H., Beurs E.D., Geerlings S.W., Tilburg W.V. (2010). The impact of depression on the well-being, disability and use of services in older adults: A longitudinal perspective. Acta Psychiatr. Scand..

[27] Johnson D. (2005). Two-wave panel analysis: Comparing statistical methods for studying the effects of transitions. J. Marriage Fam..

[28] Lu S., Wu Y.P., Mao Z.F., Liang X.H. (2020). Association of Formal and Informal Social Support with Health-Related Quality of Life Among Chinese Rural Elders. Int. J. Environ. Health Res..

[29] Courtemanche C.J., Zapata D. (2014). Does Universal Coverage Improve Health? The Massachusetts Experience. J. Policy Anal. Manag..

[30] Bernardes S.F., Matos M., Goubert L. (2017). Older adults’ preferences for formal social support of autonomy and dependence in pain: Development and validation of a scale. Eur. J. Ageing.

[31] Hao X.Q., Yang Y.H., Gao X.T., Dai T. (2019). Evaluating the Effectiveness of the Health Management Program for the Elderly on Health-Related Quality of Life among Elderly People in China: Findings from the China Health and Retirement Longitudinal Study. Int. J. Environ. Health Res..

[32] Cheng L.G., Liu H., Zhang Y., Zhao Z. (2018). The health implications of social pensions: Evidence from China’s new rural pension scheme. J. Comp. Econ..

[33] Tseng F.M., Petrie D.J. (2014). The implications for health, depression, and life satisfaction from a permanent increase in income for the disadvantaged elderly: Evidence from Taiwan. Rev. Soc. Econ..

[34] Lam W.W.Y., Loo B.P.Y., Mahendran R. (2020). Neighbourhood environment and depressive symptoms among the elderly in Hong Kong and Singapore. Int. J. Health Geogr..

[35] Kwong E., Kwok T.T.Y., Sumerlin T.S., Goggins W.B., Leung J., Kim J.H. (2020). Does subjective social status predict depressive symptoms in Chinese elderly? A longitudinal study from Hong Kong. J. Epidemiol. Community Health.

[36] Chen X., Silverstein M. (2000). Intergenerational social support and the mental well-being of older parents in china. Res. Aging.

[37] Chen F.N., Short S.E. (2008). Household context and subjective well-being among the oldest old in China. J. Fam. Issues.

[38] Koizumi Y., Awata S., Kuriyama S., Ohmori K., Hozawa A., Seki T., Matsuoka H., Tsuji I. (2005). Association between social support and depression status in the elderly: Results of a 1-year community-based prospective cohort study in Japan. Psychiatry Clin. Neurosci..

[39] Muramatsu N., Yin H., Hedeker D. (2010). Functional declines, social support, and mental health in the elderly: Does living in a state supportive of home and community-based services make a difference?. Soc. Sci. Med..

[40] You X.Y., Zhang Y.L., Zeng J.F., Wang C.J., Sun H.P., Ma Q.H., Ma Y.N., Xu Y. (2019). Disparity of the Chinese elderly’s health-related quality of life between urban and rural areas: A mediation analysis. BMJ Open.

[41] Dong X.Q., Simon M.A. (2010). Health and aging in a Chinese population: Urban and rural disparities. Gerontol. Geriatr..

[42] Tian T., Chen Y.H., Zhu J., Liu P.L. (2015). Effect of air pollution and rural-urban difference on mental health of the elderly in china. Iran. J. Public Health.

[43] Baernholdt M., Yan G.F., Hinton I., Rose K., Mattos M. (2012). Quality of life in rural and urban adults 65 years and older: Findings from the national health and nutrition examination survey. J. Rural Health.

[44] Hopman W.M., Harrison M.B., Coo H., Friedberg E., Buchanan M., Van Den Kerkhof E.G. (2009). Associations between chronic disease, age and physical and mental health status. Chronic Dis. Can..

[45] Frayne S.M., Skinner K.M., Lin H., Ash A.S., Freund K. (2004). Effect of patient gender on late-life depression management. J. Womens Health.

[46] Burckhardt C.S. (2010). The effect of therapy on the mental health of the elderly. Res. Nurs. Health.

[47] Levkoff S.E., Macarthur I.W., Bucknall J. (1995). Elderly mental health in the developing world. Soc. Sci. Med..

[48] Howden-Chapman P.L., Chandola T., Stafford M., Marmot M. (2011). The effect of housing on the mental health of older people: The impact of lifetime housing history in whitehall II. BMC Public Health.

[49] Tong M. (2010). A study on the concept of mental health and its implication for social work education in the context of chinese communities. Can. Soc. Sci..

[50] Reeves A., McKee M., Mackenbach J., Whitehead M., Stuckler D. (2017). Public pensions and unmet health need among older people: Cross-national analysis of 16 European countries, 2004–2010. J. Epidemiol. Community Health.

[51] Tavares D., Santos N., Dias F.A., Souza L.D.M. (2015). Morbidities and life quality of elderly males in rural and urban areas. Acta Entiarum Health Sci..

[52] Bai Y., Bian F., Zhang L., Cao Y. (2020). The impact of social support on the health of the rural elderly in china. Int. J. Environ. Health Res..

